# A qualitative exploration of nurses leaving nursing practice in China

**DOI:** 10.1002/nop2.11

**Published:** 2014-12-22

**Authors:** Junhong Zhu, Sheila Rodgers, Kath M. Melia

**Affiliations:** ^1^ Arthur Labatt Family School of NursingUniversity of Western OntarioLondonOntarioCanada; ^2^ Nursing StudiesSchool of MedicineHangzhou Normal UniversityHangzhouChina; ^3^ Nursing StudiesSchool of Health in Social ScienceUniversity of EdinburghEdinburghUK

**Keywords:** China, expectations, nursing turnover, nursing workforce management, power, shortage, voluntary leaving

## Abstract

**Aim:**

This paper reports a theoretical understanding of nurses leaving nursing practice by exploring the processes of decision‐making by registered nurses in China on exiting clinical care.

**Background:**

The loss of nurses through their voluntarily leaving nursing practice has not attracted much attention in China. There is a lack of an effective way to understand and communicate nursing workforce mobility in China and worldwide.

**Design:**

This qualitative study draws on the constant comparative method following a grounded theory approach.

**Method:**

In‐depth interviews with 19 nurses who had left nursing practice were theoretically sampled from one provincial capital city in China during August 2009–March 2010.

**Results:**

The core category ‘Mismatching Expectations: Individual vs. Organizational’ emerged from leavers’ accounts of their leaving. By illuminating the interrelationship between the core category and the main category ‘Individual Perception of Power,’ four nursing behaviour patterns were identified: (1) Voluntary leaving; (2) Passive staying; (3) Adaptive staying and (4) Active staying.

## Introduction

In the past three decades, Chinese nurses experienced rapid economic, social‐political and educational changes in healthcare system reforms. These changes have greatly impacted their employment decisions. Although nursing shortage in China is more serious than that in many other developed countries (WHO 2011, You *et al*. 2013), Chinese nurses continue to voluntarily leave nursing practice. They intend to keep their leaving quiet and personal, resulting in ineffective communications with policy makers. The main author initiated her PhD study on nursing mobility in 2008, when the Chinese pilot health reform started. This report aims to unfold a theoretical explanation of voluntary departure from the leavers’ perspective.

## Background

### Historical nursing employment in China

The Chinese government has established *dingbian* as a staffing legislation in the Chinese healthcare system since 1978. The staffing requirements in *dingbian* focus on the numbers of hospital staff based on the bed size of hospitals and the ratio of nurses per hospital bed is the only recommended standard (Ministry of Health of the People's Republic of China (MHPRC)'s 1978). This differs from most western countries, where the ratio of patients to nurses based on daily care ward by ward determines the nursing staffing (Rafferty *et al*. 2007, Aiken & Cheung 2008, Conway *et al*. 2008). From 1979, the Chinese healthcare system gradually transferred from a planned to a market economy (Ma *et al*. 2008). Since then, the state subsidy has been cut dramatically, from 30% to less than 7% of total hospital expenditure (Yang 2008).

The Chinese health workforce has slightly increased and has reached the WHO minimum workforce threshold since 2006. However, almost all hospitals tend strictly to limit nursing staff but increase numbers of doctors (MHPRC 2005, World Health Organization (WHO) 2006, Liang *et al*. 2007). While China is one of a few countries that have more doctors than nurses (WHO 2011), the Ministry of Health China (2005) still emphasises *dingbian* to set up a ratio of per hospital bed to nurses at 1:0·4, nevertheless it is 1:1 in OECD countries (Anderson *et al*. 2005). The historical Chinese nursing employment pattern has resulted in a far more demanding clinical situation due to the inadequacy of nursing staffing than in most industrialised countries (Zhu *et al*. 2014). A high rate of dissatisfaction and intention to leave nursing has been reported in different areas nationwide in China (Sun *et al*. 2001, Ye *et al*. 2006, Lu *et al*. 2007). Nevertheless, official statistics is lacking to monitor how many nurses have actually left nursing practice in China.

### Responses to the nursing shortage and turnover

Nursing shortage is an international problem WHO 2006); however, the literature on nursing shortage is mainly based on the US, the UK and other industrialized countries. These studies advocate that strategies for solving the problem include encouraging recruitment from a broad recruitment base, improving retention, attracting former nurses back into the profession and increasing international recruitment from abroad (Aiken *et al*. 2001, Shields & Ward 2001, Buchan 2006, Rafferty *et al*. 2007).

Reducing the turnover tends to be the main concern to meet the target number of registered nurses in dealing with the nursing shortage, although Lewis (2002) and Rafferty *et al*. (2007) warned that the shortage is not only merely about numbers but also a matter of how these numbers are most effectively deployed in the healthcare system. Most of the literature on nursing turnover uses the intention of leaving to predict actual turnover by studying the nurses who are still employed in the profession and mainly focuses on matching or testing different theoretical models by quantitative designs (Maertz & Griffeth 2004, Hayes *et al*. 2006, Takase *et al*. 2008). However, even the most extensive turnover predictive models have underestimated some important antecedents as Maertz and Campion (2004) commented. Although the reasons for the growing shortage of nurses appear similar everywhere (Tierney 2003), the relevant available comparative studies between China and the developed countries (Lu *et al*. 2007, You *et al*. 2013) have contributed little to an understanding of the nursing mobility and shortage in China.

Chinese nurses are facing unique historical, political, cultural and educational difficulties in trying to solve the problem of the nursing shortage by retaining nurses. Compared with the efforts made in most western countries, Chinese hospital managers are reluctant to admit that nurses are important to the quality of health care based on a profit‐driven system (Hsiao 2008, Zhu 2010). The Chinese Nursing Association (CNA) leadership hopes the backlash of a national nursing shortage and subsequent negative effects on patient outcomes will trigger Chinese healthcare reform and will improve the welfare of Chinese nurses (Xu 2003, Hu *et al*. 2010). Nevertheless, the nursing workforce problems were not considered relevant and important in the current pilot stage of the new healthcare reform (MHPRC 2009). Nurses voluntarily leaving nursing practice have a wealth of experience and what they have to say can help understand the current nursing workforce mobility and retention problem.

## The study

### Aim

The aim of the study is to understand why nurses leave nursing practice by exploring the decision‐making process of registered nurses who have exited clinical care in China. The research questions focus on:

- How do leavers describe their experiences of being a clinical nurse during their entering, practising and leaving nursing practice?
- How do they explain their reasons for leaving nursing practice?

### Design

The qualitative study draws on the constant comparative method following a grounded theory approach (Glaser & Strauss 1967, Glaser 1978). In‐depth interviews with 19 nurses who have left nursing practice were theoretically sampled from one provincial capital city in the east of China during August 2009–March 2010.

### Ethical considerations

Ethical approval was granted by Research Ethics Committee, University of Edinburgh, UK and the provincial Research Institution of Social Science in China. An informed choice ensured the participants to voluntarily join the study and pseudonyms and confidentiality were preserved against potential harm.

### Participants

The participants included leavers with different years of work experiences in all areas of clinical care (except mental health care) who had voluntarily left their nursing practice during the past 5 years. The first selection was carried out on a wide range of leavers according to their educational and socio‐economic background, clinical practice and leaving experiences. Based on the previous interview data analysis, the researchers decided who was the next to talk with, listen to, query or observe based on a given issue important to the research (Glaser & Strauss 1967). The details of participants selection following the principle of theoretical sampling in the study have been discussed elsewhere (Zhu *et al*. 2014).

### Data collection

The interviews were audio‐recorded and field notes were made after each visit or phone call to potential participants during sampling. Meanwhile, we not only treated all the relevant literature as secondary data but also included the data naturally available to the research field, such as informal conversations or observational notes made during the research process, relevant nursing policies, hospital documents, work or personal diaries, news reports, work contracts.

### Data analysis

All interviews were transcribed verbatim and kept in their original forms for the researcher to check the accuracy of interpretation and translation during different stages of analysis and presentation by constantly comparing pieces of data, back and forward between two languages. Plausible suggestions were sought in approaching sampling according to the categories and incidents which emerge from the data by a constant comparative analysis (Glaser & Strauss 1967). Refinement of the hypothesis involved continuously reviewing the core category for internal consistency and logic by looking for negative evidence or deviant cases until it accounts for all known cases without exception (Glaser & Strauss 1967, Silverman 2010).

### Rigour

For the rigour in all phases of the research process (Zhu 2012), we applied reflexivity to examine the possible bias throughout the design and conduct of this study, adding trustworthiness to this study as suggested by Hertz (1997). The rationale behind presenting the original descriptive data here is to provide enough evidence to illustrate the concepts, and the more comprehensive data could be accessed elsewhere (Zhu 2012). According to Bryman (2004), who emphasised that enough detail about findings should be provided to enable readers to determine applicability and Glaser and Strauss (1967) who suggested that participants should be able to perceive the story as a reasonable explanation of what is going on even if it is not the case that every detail quite fits their cases, we provided the conceptual categories, the storyline and the hypothesis of theoretical model to participants, as well as the clinical nurses and nursing managers, and asked them to comment on how well the theory seems to fit their perception.

## Findings

**Figure 1:**
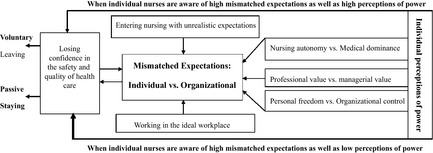
The storyline of mismatched expectations towards voluntary leaving.

This report presents the findings according to the conceptualized categories and their subcategories that emerged from the data analysis (Table 1, Figure 1), which offer suggestions about what to look for based on reality as it exists. The core category ‘Mismatched Expectations: Individual vs. Organizational’ emerged from the following three subcategories.

**Table 1:** Summary of the conceptualized categories.

Main categories	Subcategories	Themes
Mismatched Expectations: Individual vs. Organizational	Entering nursing with unrealistic expectations	Choosing nursing with collective expectations Restricting realistic expectations of nursing in education
	Working in the ideal workplace	Entering the ideal workplace Committing to the organization Struggling with a professional identity
	Losing confidence in the safety and quality of health care	Perceiving the risk in clinical practice Recognizing the organizational barriers to safety Failing to meet expectations of patients
Individual Perceptions of Power	Nursing autonomy vs. medical dominance	Comparing rewards with doctors Struggling with medical dominance
	Professional value vs. managerial value	Emphasising nurses as replaceable labour Losing enthusiasm in promotion Struggling to meet career progress
	Personal freedom vs. organizational control	Lack of reasonable nursing mobility Limited maternity leave and sick leave

### Mismatched expectations: individual vs. organizational

#### Entering nursing with unrealistic expectations

This subcategory emerged from the retrospective experiences of participants who explained how different generations chose nursing studies in the past three decades from 1978–2008. Students before 1995 received free nursing education and were more concerned about reducing the family financial burden. They usually entered nursing school at the ages of 15–16 and had better school performance. They constitute the current main nursing workforce and the majority of nursing leaders in China. This group of participants were particularly dissatisfied with the strict learning requirements of pre‐ and postregistration nursing education, as they expected that the time and energy invested in their nursing education could help their personal and professional development, while the later self‐funded generation of nurses were more concerned about accessing health care for their families.

In 1995, Chinese higher educational institutions were encouraged by the government to receive tuition fees from the students instead of offering free higher education, which was previously fully supported by the government. Since then, several strategies have been adopted to attract more students into different levels of nursing education.

First, many participants felt that the reality of being a nurse was deliberately blurred in the nursing recruitment process. They enter nursing with a vague idea of the demands of the job:

The benefits written in the recruitment document sounded pretty good. It talked about nursing research, nursing education and nursing management, but it did not say that you would work as a clinical nurse. (Rao)

The majority of participants said that they were transferred into nursing studies without true desire to be nurses. While different levels of education between the universities and colleges became fiercely competitive with a market orientation, Chinese students are required to accept the pre‐condition for getting a university admission offer ‘I agree to be transferred to other subjects’. Although nursing is an unwelcome career choice in current Chinese society, involuntarily transferring students into nursing studies from other subjects has been taken for granted. As a nursing teacher now, Yuan thought that a lack of proper support for students who involuntarily entered nursing study has a negative impact on students’ learning attitudes and their leaving the nursing practice early. She talked about the high ratio of those of leaving nursing among her previous classmates who graduated from a top Chinese university:

There were 30 classmates in my class, only 5–6 of them are still working as nurses, 10 became doctors and the others became nursing teachers. One of the classmates graduated with her master's degree in medicine and went back to work in nursing, but she soon left and worked abroad. (Yuan)

These leavers who became doctors, nursing teachers or worked abroad usually were excellent graduates with a bachelor's degree or above, who were regarded as student role models in current Chinese nursing education. Therefore, their leaving from nursing clinic has a negative impact on nursing morale.

Second, while the bachelor nursing education programme is facing a great recruitment challenge, the colleges set relatively low entrance requirements for nursing. However, as part of the one‐child generation, many current nursing students regard entry to nursing as the key to accessing better job opportunities:

I found it was very difficult to survive without a college degree. …I am not interested in nursing, but no matter what, I must accept the offer and complete the study for a degree first. (Ming)

Whether actively or passively choosing nursing, the realistic expectations of being a clinical nurse were surprisingly omitted by all participants during the admission. Currently, Chinese nurses are predominantly women from the huge rural population with farming or lower working class backgrounds:

It is true that very few city residents would send their children to nursing school; however, in the rural areas, the students… their biggest dream is to work in the cities. … So don't worry. There are so many people available. (Ling)

The view is popular that there is no problem to recruit nursing students in China. The historical unrealistic expectations of nursing were not only created in nursing education but also extended to the hospital recruitment process.

#### Working in the ideal workplace

The Grade Three hospitals were regarded by participants as the ideal workplace for nurses with different levels of nursing education. Working in these hospitals means a stable job with a decent income and better opportunities for personal and career development. It is difficult for nurses with low‐level education to find a permanent nursing job with a fair contract, resulting in a high rate of unemployment among nursing college leavers. The rigorous recruitment selection process has reinforced a strong organizational commitment among the successful recruits:

As it was not easy to get the job in the hospital, when I started to work in the hospital, I never thought that I would leave the hospital. I was determined to do my job well until retirement. (Xue)

All participants appreciated the opportunity to work in these hospitals and expected to do a good job at the outset. However, the well‐educated and qualified nurses had a heavy workload without clear role boundaries and effective skill‐mixed teamwork:

Nurses are responsible for informing patients about fee collection, which should not be their duty. … It is terrible when the responsibilities are not clear…I mean when you try to work well, it will become your extra daily work and your responsibility. … It is difficult for nurses to forget what they could do well for patients. Your good intention is to help patients, but when there is any problem caused by such situations, the nurses are often blamed. (Chun)

Many participants said that the different processes connected with the quality of health care for patients are fully understood by nurses. They adopted different strategies to ensure effective care, but the unbearable workload and shortage of staff have negatively affected the well‐being of nurses.

#### Losing confidence in the safety and quality of health care

Although the nurses’ initial expectations of nursing appear to be met by being successful in getting a job in their ideal work place, an increasing awareness of the high risk of clinical practice negatively impacted on many participants’ faith in nursing (Zhu 2012). The authors have reported elsewhere that the ideal workplace could not support individual nurses to achieve their professional values and ideals of nursing and all participants, both experienced and inexperienced, lost confidence in the safety and quality of health care (Zhu *et al*. 2014). Work‐related stress was reported to be related to suicide, depression, fatigue, sleep disorder, nervousness, anxieties and sadness. The evidence of physical stress and emotional or moral distress among clinical nurses was also frequently reported, which in turn negatively influenced their professional identities and work enthusiasm (MacKusick & Minick 2010, Kelly 2012, Zhu *et al*. 2014).

The above three subcategories describe mismatched expectations of nursing between individuals and organizations and the following three subcategories further explain how the participants adjusted their employment behaviour and made a career decision towards voluntary leaving according to their ‘individual perceptions of Power’ (Figure 1).

### Individual perceptions of power

#### Nursing autonomy vs. medical dominance

Participants, particularly those who entered nursing practice before the year 2000, expressed that ‘the relationship between nurses and doctors was not as good as stated earlier’:

Doctors want to make more money! So many patients received unnecessary IV at midnight…Sometimes I got very angry…I was scared that I might lose control of carrying out all of the tasks. (Xia)

One of the common complaints about doctors from nurses was that they had to accept an unnecessarily high workload due to doctors prescribing excessive medication and treatment. Although Xia was sympathetic to patients and angry at doctors, she only linked her leaving to the workload. These situations have caused an ethical dilemma for nurses. They generally felt difficulty in dealing with it under medical dominance:

It will put the nurse in a difficult situation. Nursing managers often emphasize that we should not do ‘good deeds’ and we must call doctor if anything happened to patients. We all need clear awareness of self‐protection. No one dares do the same thing as before. (Chun)

When nurses were afraid of hurting their personal or work relationship with doctors by taking a stand against unnecessary treatment, their ability to protect patients’ rights was sorely weakened.

#### Professional value vs. managerial value

Participants’ leaving experiences confirmed the popular view among nursing management that individual nurses’ leaving is not a problem for the hospital but for the individuals:

Actually they did not care to lose nurses; there are plenty of young nurses available to replace us. (Yun)

All participants were disappointed at the lack of managerial support for the retention of nurses in clinical practice. They thought that the nursing managers and hospital administrators did not value experienced nurses’ work by considering nurses as replaceable labour.

Meanwhile Chinese nurses are required to pass regular examinations and spot tests and fulfil publication requirements for their career progress, which was expected to improve the quality of care. However, many participants thought that meeting these managerial values for nursing does not ensure that nurses can carry out standard practice under the heavy workload with the time and staff constraints.

#### Personal freedom vs. organizational control

Many participants expressed that there is a lack of reasonable nursing mobility in the Chinese healthcare system:

Very few nurses could get permission to move in the hospital. The director of nursing thought that it was a tough task to transfer nurses between units since there were too many nurses with similar requests and she was too tired to deal with such requests every day. Therefore, she decided to refuse all the requests. My heart sank when I heard that I had to go back to the ICU from the nursing office, but I knew that I had to go back. (Bo)

Chinese nurses do not have autonomy to choose the most compatible nursing practice environment. Meanwhile hospitals also use administrative power and financial punishment to prevent nurses leaving:

If you ask to leave the hospital within five years, you must pay 15,000 yuan, or 5000 yuan after you have worked over 5 years, since you breach the contract. The terms are solely added for the benefit of the hospital.…Actually I did not want to pay the 15,000 yuan penalty for leaving, but my parents said that I should not offend the hospital, as the hospital is an influential institution which might negatively influence my future. (Yan)

Nurses had to accept penalties for leaving, which was reinforced by the hospital employment contracts. When the participants lost hope of achieving their individual expectations, the ideal workplace became a trap for some nurses who intended to leave but could not afford the cost of leaving. Meanwhile, threats of firing and financial punishment have been used by the managers to limit sick leave and maternity leave. Many complained that there was a lack of family friendly policies for nurses in hospitals. Concerning the reality of struggling with work and family conflicts in the strict maternity and sick leave regulations, nearly all the participants had support from their family in their leaving decision to improve their well‐being and quality of family life. The participants felt more confident than their colleagues who are unable to empower themselves to leave. Leaving nursing practice was interpreted by them as a way to pursue personal freedom.

## Discussion

**Figure 2:**
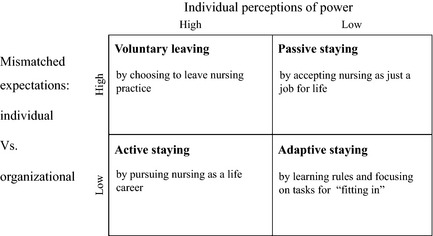
Mismatched expectations model.

The interrelationship between the ‘Mismatched expectations: individual vs. organizational’ and ‘Individual perception of power’ was further illuminated through a cross‐tabulation (Figure 2). Glaser (1978:64) suggested that, when the analyst uses relevant and grounded data‐determined distinctions with logical elaboration in a cross‐tabulation, it ‘helps achieve the goal for theory of parsimony of concepts, while at the same time richly densifying the theory’; however, caution needs to be raised that the aim of parsimony of theory may lose some explanatory power in the conceptual categories. We agree that the importance of study is not to discover a theory, but to assist in understanding the issues under investigation (Heath & Cowley 2004). Therefore, the cross‐tabulation in this study was used as an analytical device, serving as a strategy for handling data and understanding the issues under investigation.

Based on the data‐determined ‘high’ and ‘low’ distinctive dimensions of main categories, the cross‐tabulation reduced the continuing nursing career behaviours and consequences of nursing employment decision into four typologies, including Voluntary Leaving, Active Staying, Adaptive Staying and Passive Staying (Figure 2)**.** The cross‐tabulation further suggests following hypotheses to understand Voluntary Leaving:

- The higher the degree of mismatch between individual and organizational expectations of nursing recognized by the nurses and the greater the extent of imbalance of power the individual nurses perceived, the more likely it is that the nurses intend to leave the powerless status of being a clinical nurse in the organization.
- The more difficult it becomes for the nurses to achieve their individual expectations by exercising nursing autonomy in their nursing career, the more likely it is that they actually empower themselves to leave nursing practice.

It also suggests that nurses chose an active staying in their nursing career when the individual and organizational expectations of nursing were more aligned and the individual nurses were able to exercise nursing autonomy to achieve their professional values.

The hypotheses developed from this study support the argument that the reason why nurses entered the profession should be considered in association with the reasons why nurses leave (Duffield *et al*. 2004a,b). Duffield *et al*. (2004b) optimistically recommended choosing nursing as a stepping stone to future careers, since they found that nurses who were well qualified and skilled were capable of making the transition to a range of other careers. However, the Mismatched Expectations model suggests that the recommendation of Duffield *et al*. might attract more students into nursing to ensure the survival of the educational institution, but it would inevitably increase unrealistic expectations of nursing when nurses were inspired to choose nursing as a stepping stone for other careers. The findings indicate current Chinese nursing enrolment and education strategies have not effectively helped the nursing students to clarify and establish realistic expectations of nursing.

When ‘mismatched expectations: individual vs. organization’ emerged as the core category from the data, it was noteworthy that there is little consensus in current literature on how expectations are best defined from sociological, psychological, organizational and nursing perspectives. The definition of ‘role expectation’ provided by Biddle (1979:256) makes a useful starting point to examine the expectations of nursing both for individuals and organizations: ‘expectations that are structured for the roles of position within a social system.’ Nevertheless, ‘mismatched expectation: individual vs. organizational’ in this study is beyond a static concept of role expectations or professional expectations of being a nurse within a broad educational, economic and social context in China.

The two subcategories ‘entering nursing with unrealistic expectations’ and ‘working in the ideal workplace’ coincide with the two main concepts of pre‐entry expectations and postentry experiences in the Met Expectations model developed by Porter and Steers (1973). However, our study does not support this model, which agrees that the confirmation of pre‐entry expectation concerning jobs leads to low levels of voluntary turnover (Wanous *et al*. 1992, Irving & Meyer 1994). The third subcategory ‘losing confidence in the safety and quality of health care’ suggests that, although the pre‐entry expectations appear to be met by nurses’ successful entries into the ideal work place, the gradually increasing awareness of the high risk of clinical practice has a negative impact on many participants’ faith in nursing. This is because it goes against their expectations of taking professional accountability for the safety and quality of care, which is regarded as the essential value of being a good nurse (Zhu *et al*. 2014).

The expectancy‐value model was developed from the expectancy theory in psychology to predict job turnover and satisfaction (Vroom 1964, Baard *et al*. 2004). It also suggests that, when individuals can exercise a degree of personal control over their intrinsic value of work, they are more likely to be perceived as experiencing job satisfaction (Baard *et al*. 2004). We found that this connection is helpful to validate the core category in understanding the importance attached to safety and quality of health care for the participants, which does shape their responses to dissatisfaction and stress and orientate their career attitude and behaviour during their nursing practice. The study also suggests that the leaving decision was determined neither simply by the individual commitment to caring (McGrath 2006), nor safety (Smith *et al*. 2009). It was an interactive decision‐making process, which depended on whether the individual and organizational expectations of nursing were aligned towards the safety and quality of health care.

Lait and Wallance (2002) identified ‘unmet expectations’ as critical in explaining job stress and suggested that the passage of time may result in changes in ‘professional's expectations,’ but the data were limited by their cross‐sectional design (Lait & Wallance 2002). Maben *et al*. (2006, 2007) found that the mismatches between nursing as taught and as practised have a profound impact on morale, job satisfaction and intention of leaving nursing. This view was fully supported here and in turn validated the concept of Mismatched Expectations that has focused on the interactive expectations between organizations and individuals.

The category ‘individual perception of power’ was integrated into the Mismatched Expectations model, which further explains why there was a relatively higher rate of dissatisfaction and intention to leave among Chinese nurses (Ye *et al*. 2006, Lu *et al*. 2007) but a particularly low rate of actual leaving.

Currently there is little literature reporting how the dynamics of power or powerlessness relate to nurses’ leaving. The term ‘power’ has been defined in politics, economics, management and sociology as the idea of ‘power over’ and the ‘command‐obedience relationship’ (Weber 1978, Lukes 2005). Nurses are frequently described as a powerless workforce and current nursing literature proposes that nurses should be empowered by nursing managers (Kanter 1993, Laschinger *et al*. 1999, Bradbury‐Jones *et al*. 2008, Ning *et al*. 2009). However, our findings suggest that nursing managers lack the ability to empower their staff, which further oppressed their own professional value and nursing autonomy. As Chavasse (1992:2) declared, ‘No‐one can value others unless they value themselves’. Our findings concur with the argument that the term ‘empowerment’ is overused in its application to nurses without a full understanding of nurses’ perception of power, both in their working and non‐working domains (Gilbert 1995, Leyshon 2002, McGrath 2006).

The category of ‘individual perception power’ has clarified three dimensions of power in the nursing work environment that individual nurses perceived, all of which is influenced by their reaction to key groups in their nursing practice in their work and personal life contexts: ‘Nursing autonomy vs. medical dominance’, ‘Professional value vs. managerial value’ and ‘Personal freedom vs. organizational control’. The findings indicate that the evaluation for promotion and nursing career progress following medical science criteria did not properly value and reward the great efforts the nurses made for the quality and cost efficiency of health care, which tends to undermine their professional value of nursing. This study supports the view of McGrath (2006) that nurses lost ground concerning power if they lost confidence in the value of what they do.

As the Mismatched Expectation model indicates, the two behaviour patterns, adaptive staying and passive staying, are characterised by low individual perception of power. Adaptive staying has concordance with the concept of ‘fitting in,’ which was first described by Melia (1981). It suggests that, when students cannot integrate education and service segment in their transient stage, they learn how to go through their educational and clinical training separately. Attree *et al*. (2008) also found that feelings of being vulnerable and their need to fit in made students feel unable to challenge unsafe practice. Our study further confirms that, when new nurses could not receive proper support from clinical supervisors and managers, they continue to adapt themselves to survive as their priority by learning organizational rules and focusing on tasks. MacKusick and Minick (2010) also found that feeling a lack of support as a new nurse was a reason nurses cited for leaving clinical practice even years later.

The extent of individual perception of power might still be low when nurses continue to practise over time. When they realise a high degree of mismatched expectations between individuals and the organization over their practice under medical dominance without managerial and organizational support, they compromise their individual expectations of nursing to meet the organizational expectations and accept nursing just as a job for life. The evidence indicates that the nurses with lower perception of power do not have the freedom to leave nursing practice, but loose enthusiasm and commitment in their nursing career. In this way, they are engaging in the passive staying behaviour pattern.

It is understandable that the low rate of nurses’ voluntarily leaving nursing practice could be temporary under the current Chinese organizational control both from educational and hospital settings. However, nurses’ personal freedom will likely increase in the future due to demographical, financial, educational and social changes in China, which means if nurses are unable to exercise nursing autonomy to achieve essential nursing values, they are more likely to challenge the mismatched expectations and pursue freedom by leaving nursing. Therefore, if the policy makers and the hospital managers still take organizational control for granted without effective intervention, the rate of voluntary leaving may dramatically increase.

## Conclusion

The study indicates that ineffective nursing employment may not only occur when experienced nurses choose voluntary leaving but also happens when they resort to passive staying and when novices continue an adaptive staying mode and act towards patients with a dehumanized attitude. The Mismatched Expectations model suggests that the higher the degree of mismatch that the nurses recognised between individual and organizational expectations of nursing and the more difficult it becomes for the nurses to achieve their individual expectations by exercising nursing autonomy with managerial and organizational support in a nursing career, the more likely it is that they empower themselves to leave nursing practice. This study is restricted by the reality of the difficulty in expanding the data collection from different populations and settings following the theoretical sampling in a time limited PhD project. Nevertheless, the theoretical perspective may contribute to the international debate on effective retention strategies towards effective nursing workforce management. The hypotheses and suggestions warrant verification and readily modification in nursing workforce management in different healthcare systems by further research.

## Conflict of interest

The authors declare that there is no conflict of interest with the funders.

## Author contributions

All authors have agreed on the final version and meet at least one of the following criteria [recommended by the ICMJE (http://www.icmje.org/ethical_1author.html)]:

- substantial contributions to conception and design, acquisition of data, or analysis and interpretation of data;
- drafting the article or revising it critically for important intellectual content.
